# A novel deep-learning approach for robust identification of plant diseases

**DOI:** 10.1371/journal.pone.0353374

**Published:** 2026-07-16

**Authors:** Justin Hoffmann, Christopher Mai, Ricardo Buettner

**Affiliations:** Chair of Hybrid Intelligence, Helmut-Schmidt-University/University of the Federal Armed Forces Hamburg, Hamburg, Germany; Bina Nusantara University, INDONESIA

## Abstract

Rising temperatures and changing weather conditions are accelerating the spread of plant diseases and increasing the threat to global food security. Reliable detection of leaf diseases is therefore essential to protect crop yields and ensure food quality. Deep learning has proven to be a powerful tool for classifying leaf diseases across various crops. Due to the natural variability of plants, plant diseases often appear in irregular structures. Surface unevenness, folds, or dirt particles are common in field images and can be mistakenly identified as important features by convolutional neural networks (CNNs). This is a challenge that has not been sufficiently addressed in previous studies. This study proposes a novel deep learning approach that takes into account both the specific visual characteristics of plant diseases and potential disturbances in the microstructure, such as surface irregularities or prominent leaf veins, which may mislead the model. Using stratified five-fold cross-validation on a peer-reviewed dataset, which comprises 2,801 images of radish leaves across five classes (healthy, three disease classes: mosaic virus, black leaf spot, and downy mildew, and one pest-affected class: flea beetle), the proposed method achieved an average and balanced accuracy of 99.86%, establishing a new dataset-level benchmark in the field and demonstrating its effectiveness. The results indicate that the proposed approach may provide a promising basis for future applications in agricultural field monitoring, automated sorting and post-harvest quality control, offering potential to reduce both food waste and associated costs.

## Introduction

Climate change is exacerbating the global threat to food security, as changing weather conditions, cultivation environments, and the associated increase in plant diseases and pest infestations can lead to significant crop failures [2–5]. These crop failures can have substantial economic and social consequences [6,7]. Since plant health is often reflected in leaf appearance, visual inspection plays a key role in disease assessment [8]. However, manual inspection of all plant leaves is unrealistic and inefficient across large-scale cultivation areas [9]. The precise automated identification and classification of plant leaf diseases through drones or tractors using a deep learning approach thus represents a logical and urgent application [8,9]. Thus, an accurate classification of infected leaves could enable automated sorting processes that determine which produce meets market quality standards and which must be excluded [10]. This capability may help to reduce post-harvest losses, improve economic efficiency, and uphold food safety [10]. Deep learning approaches have already demonstrated good performance across a wide range of plant leaf disease classification tasks. Studies have reported high accuracy in classifying plant leaf diseases in various crops [11–20]. Plant species vary greatly in terms of shape, color, and leaf structure. The same applies to plant diseases, which can manifest in different forms and intensities. In addition, there are various interfering factors, such as surface irregularities, differences in leaf texture, lighting conditions, or dirt particles. These real-world conditions can cause CNNs to focus on misleading features and make incorrect predictions.

Several studies [16,21,22] have shown that preprocessing filters can be used to address this by reducing visually irrelevant image information before classification. Buettner et al. [23] demonstrated that a carefully chosen preprocessing filter, such as a Gaussian filter, can significantly improve accuracy in industrial defect detection by reducing irrelevant features and enhancing defect visibility. This insight into the Gaussian filter could be used to classify radish diseases by highlighting important defective areas and disregarding less relevant areas. Other studies [24,25] have also used Gaussian filtering as a preprocessing step for the classification and detection of plant leaf diseases. Although previous studies have already achieved valuable advancements, to the best of our knowledge, no study has yet combined a CNN with Gaussian filtering and evaluated this combination in the specific context of radish plant leaf disease classification. Additionally, a large proportion of studies in this thematic domain [26,27] rely on datasets with limited public availability or incomplete documentation. Using such datasets can make it difficult to assess label quality, annotation procedures, and dataset composition. Furthermore, the evaluation of methods is frequently insufficient, leaving the stability and reliability of the approaches unclear. To address this research gap, we present a novel deep-learning approach that combines an established CNN with an established preprocessing filter into a combination designed for local pattern recognition, enabling the detection of plant-leaf disease symptoms. Compared to other preprocessing methods, the Gaussian filter demonstrated the highest accuracy. To demonstrate its stability and reliability within the dataset, which is often not addressed in other studies, the approach is evaluated using stratified 5-fold cross-validation. This method was applied to ensure a consistent class distribution across all folds, since even minor class imbalances or small variations in class distribution between folds can affect model evaluation. It also provides a more robust performance estimate by reducing sensitivity to a single data split. Due to its important role in the economy and as a food source, particularly in Asian countries, we evaluate our novel approach using a publicly available and well documented radish dataset from a peer-reviewed source [1] containing five classes and a total of 2,801 images.

The results demonstrate that our novel approach delivers robust and consistent performance, establishing a new benchmark within the evaluated dataset and comparison setting with an average accuracy and balanced accuracy of 99.86%. In a contextual comparison with related work, this corresponds to a reduction in the error rate. A lower error rate is particularly important for plant disease recognition, as it could reduce undetected infections that may cause yield losses and could minimize false alarms, thereby avoiding unnecessary pesticide use. By demonstrating reliable classification of radish plant leaf diseases across all critical performance metrics, the proposed approach of this dataset-level engineering study establishes a promising foundation for future deployment-oriented evaluation. By enabling precise and robust disease detection within the evaluated dataset, this work contributes to sustainable yield protection, to food security, and economic resilience in radish production. The main contributions of this work can be summarized as follows:

1) We present a novel deep learning approach that addresses the specific challenges of plant disease classification and establishes a new dataset-level benchmark through its robust performance.
2) We demonstrate that a carefully selected preprocessing filter can lead to the highest classification accuracy without increasing model complexity.

This paper is structured as follows: Section Related Work provides an overview of related work, including the problem domain, advances in CNNs, and previous approaches to radish plant leaf disease classification. Section Methodology presents the methodology, detailing the model architecture, Gaussian filter, training procedure, dataset characteristics, evaluation metrics, and the setup used. Section Results reports the experimental findings. In section Discussion, we interpret the results and discuss practical implications. Section Conclusion concludes the study, addresses its limitations, and outlines directions for future research.

## Related work

In recent years, deep learning has gained traction as a highly effective method for plant leaf disease classification. Numerous studies have reported high classification accuracies [28], with several exceeding 97% on crops such as tomato, rice, maize, beans, apple, and many more [11–20]. Many approaches leverage transfer learning with pretrained CNNs, including ResNet, Inception, and EfficientNet [11,18,19,28]. To further enhance accuracy, some studies incorporate attention mechanisms, transformer-based components, or preprocessing filters [13,15,17–19]. Radish (*Raphanus sativus*) is a widely cultivated vegetable crop, particularly in Asian countries, and plays an important role in human nutrition, regional agriculture, and economies [29]. However, radish plants are susceptible to a variety of leaf diseases and pests, including Mosaic Virus, Black Leaf Spot, Flea Beetle infestation, and Downy Mildew [1]. Given the nutritional, agronomic, and economic importance of radish crops [29], the accurate identification of leaf diseases is crucial to safeguard their sustainable productivity.

### Radish plant leaf disease classification

Radish plants are a valuable food source for humans and also represent an important export product, especially in Asian countries where they hold economic relevance. Detecting diseased leaves serves as a preventive step to remove affected produce and help prevent the spread of disease to other plants. Quoc et al. [30] proposed SCOLD, a vision–language model for leaf disease classification that leverages contrastive learning between image–text pairs and a context-aware soft target (CST) mechanism to improve generalization. The architecture integrates Swin-T as the visual encoder and RoBERTa for textual representations, which was pretrained on the LeafNet dataset [30]. Their approach, SCOLD, achieved an accuracy of 95.82% in the few-shot (16-shot) setting and reached 94.37% after fine-tuning. The study by Banerjee et al. [26] introduced a hybrid deep learning approach for radish leaf disease classification. They combined a CNN comprising four convolutional layers, four max-pooling layers, and one fully connected layer, with a support vector machine (SVM) as the final classifier. The model was trained on a curated dataset of radish leaf images spanning five disease classes. The CNN–SVM architecture achieved an accuracy of 92.00% and a weighted F1-score of 81.45%. In another study, Ji et al. [27] proposed a custom hybrid deep learning model for radish leaf disease detection based on semantic segmentation. They combined a convolutional encoder with a transformer-based decoder enhanced by a hybrid attention mechanism that integrates both spatial and channel attention modules. To address class imbalance and improve segmentation performance, the architecture incorporates a hybrid loss function composed of cross-entropy and Dice loss. Evaluated on a six-class radish leaf disease dataset, the model achieved 91.00% accuracy, 93.00% precision, and 89.00% recall.

The identified publications are summarized in Table 1, detailing the classification classes, reported performance, whether the dataset was publicly available and described in a peer-reviewed source, and whether cross-validation was applied as a validation method. The aforementioned studies provide the current contextual reference point for radish plant leaf disease classification, with reported accuracies of up to 94.37%. Although the reported results represent a meaningful achievement, they also indicate that there is still potential for improvement. Additionally, plants often exhibit interfering factors such as surface irregularities, variations in leaf texture, lighting conditions, or dirt particles that can negatively impact the performance of CNNs in disease recognition. Previous studies across different image-classification domains [16,21–23] have shown that preprocessing filters can improve classification performance by potentially reducing visually irrelevant image information before classification. However, none of the reviewed radish plant leaf disease studies evaluated whether the combination of a preprocessing filter and a CNN can improve CNN-based classification accuracy in this specific domain. Furthermore, only Quoc et al. [30] utilized a publicly accessible and well-documented dataset, while the datasets used by Banerjee et al. [26] and Ji et al. [27] were either self-curated or insufficiently documented. This lack of standardized and transparent data sources limits the reproducibility and comparability of their findings. Additionally, none of the reviewed works employed cross-validation to assess the stability of model performance across data splits, which reduces the reliability of the reported results, particularly given the class imbalances and heterogeneity that commonly characterize agricultural image datasets. Without such validation schemes, the reliability of the models to unseen data remains uncertain.

**Table 1:** Overview of related work on radish plant leaf disease classification. For contextual comparative purposes, the results of this study are also included.

Reference	Year	Multiclass Classification / Classes	Accuracy	Peer-Reviewed	Cross-Validation
Quoc et al. [30]	2026	(1) Fresh Leaf (2) Mosaic Virus (3) Black Leaf Spot (4) Flea Beetle (5) Downy Mildew	94.37%	✓	✘
Banerjee et al. [26]	2024	(1) Alternaria Blight (2) White Rust (3) Root Rot (4) Mosaic Virus (5) Radish Phyllody	92.00%	✘	✘
Ji et al. [27]	2025	(1) Downy mildew (2) Black spot (3) Anthracnose (4) Bacterial black spot (5) Black rot (6) Viral disease	91.00%	✘	✘
**This study**	**2026**	**(1) Fresh Leaf** **(2) Mosaic Virus** **(3) Black Leaf Spot** **(4) Flea Beetle** **(5) Downy Mildew**	**99.86%**	✓	✓

To address these challenges, we introduce a novel deep-learning approach that combines an established CNN with an established preprocessing filter into a task-specific combination designed to capture fine-grained local patterns. This task-specific combination is intended to support the detection of visual symptoms of plant diseases that might otherwise be difficult to distinguish, thus to improve accuracy. To account for potential interfering factors on leaf surfaces, we integrated a Gaussian filter into our approach as a preprocessing step. This filter has been shown to improve accuracy in the field of defect detection across various objects by reducing irrelevant features and enhancing the visibility of the actual defect [23]. To demonstrate the stability of model performance across data splits, which is often not considered in related studies, the approach is evaluated using stratified 5-fold cross-validation on a publicly available and well documented dataset from a peer-reviewed source.

## Methodology

### Model architecture

**Fig 1:**
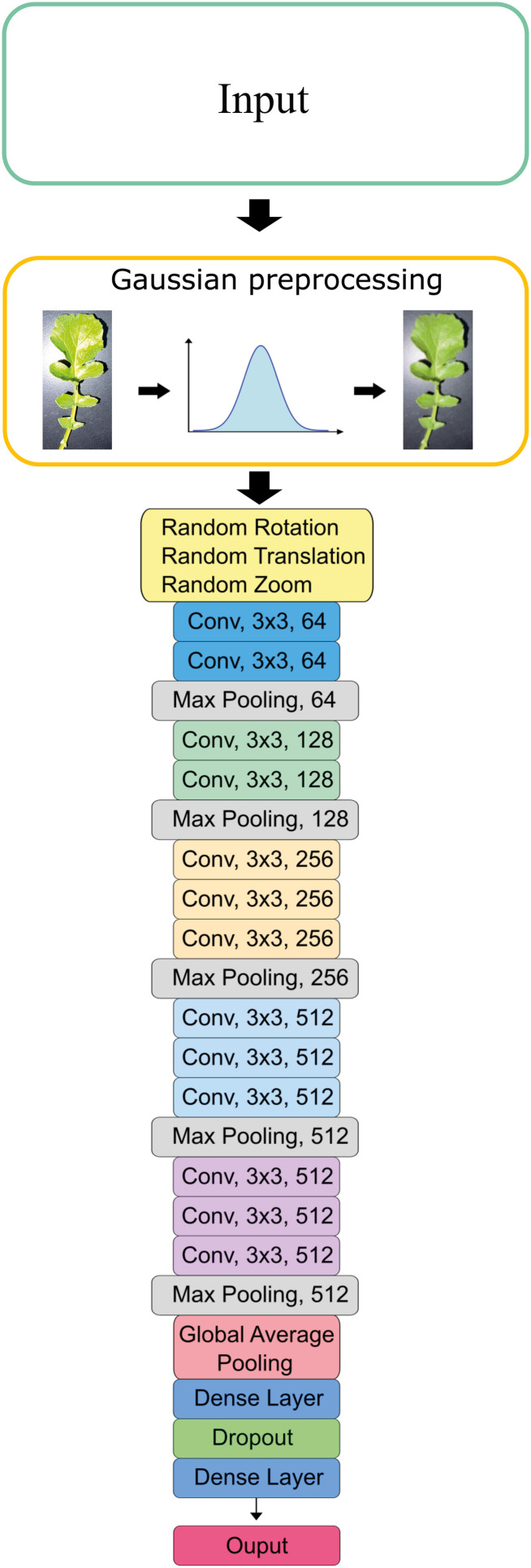
Graphical representation of the adapted VGG16-based architecture used in this study. The input images are first preprocessed using a Gaussian filter, followed by various data augmentation techniques before being passed into the model. The classification head is customized and includes two dense layers with a dropout layer in between.

Due to the inherent characteristics of plant diseases, which can sometimes present with subtle visual symptoms, a VGG16-based model was chosen. Its consistent use of 3×3 convolutional layers makes it particularly effective at capturing local patterns and textures. The overall structure of our approach is shown in Fig 1. The input images are resized to 150×150 pixels and preprocessed with a Gaussian filter to reduce unwanted noise, such as dust, dirt, or uneven lighting and enhance disease-relevant features. Afterwards, data augmentation is applied to increase training variance. Three augmentation techniques are used: random rotation, random translation, and random zoom. Their parameters are optimized through hyperparameter tuning. The preprocessed and augmented images are then passed into the model. VGG16 is a deep CNN with 16 weight layers (13 convolutional layer and 3 fully connected layer). It uses 3×3 kernels, stride 1, and one-pixel padding, relying entirely on small kernels [31]. The convolutional layers are arranged in five blocks, each followed by a 2×2 max pooling layer with stride 2 [31]. The number of feature channels starts at 64 and doubles after each pooling step, reaching 512 in the deeper layers. The original classification head is replaced by a custom one. It starts with global average pooling, followed by a fully connected layer with a tunable number of neurons and ReLU activation. A dropout layer helps reduce overfitting. Finally, a second fully connected layer with five neurons and a softmax activation generates the output.

### Gaussian filter

A Gaussian low-pass filter smooths images by removing noise, thus reducing high-frequency components [32]. The Gaussian filter used is based on a two-dimensional Gaussian function that is shown in Equation 1 [32]. The Gaussian function is used to calculate the transformation applied to each pixel in the image [33]. In the Gaussian function, x denotes the distance from the origin in the horizontal axis, while y denotes the distance from the origin in the vertical axis [32]. The symbol σ denotes the standard deviation [32]. By applying the Gaussian filter to the image, a kernel is created by sampling the Gaussian function at various distances from the center point of the distribution [32]. The extent of blurring applied to the image depends on both the size of the kernel and the σ of the Gaussian function.


$$\begin{array}{c} G(x,y)=\frac{1}{2\pi \sigma^{2}}\exp\left(-\frac{x^{2}+y^{2}}{2\sigma^{2}}\right) \end{array}$$ (1)

**Fig 2:**
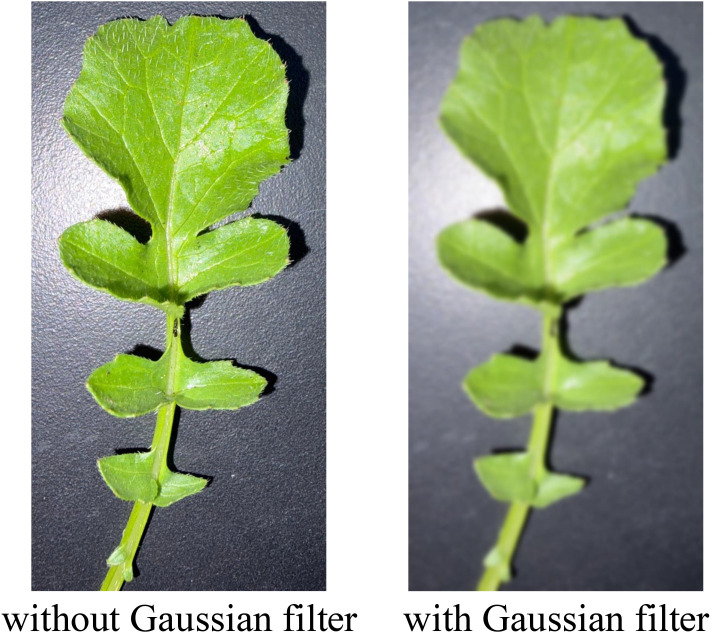
Graphical demonstration of a plant leaf image (own image) before and after applying the Gaussian filter, shown for visual comparison.

In our model, we applied OpenCV’s cv2.GaussianBlur() function to the input images, using a kernel size of 7×7. The standard deviation σ in both the x and y directions was set to 0. A σ value of 0 indicates that it is automatically computed based on the kernel size [34]. The result of using the Gaussian filter with the above settings for σ and kernel size is demonstrated in Fig 2.

### Process of training

**Fig 3:**
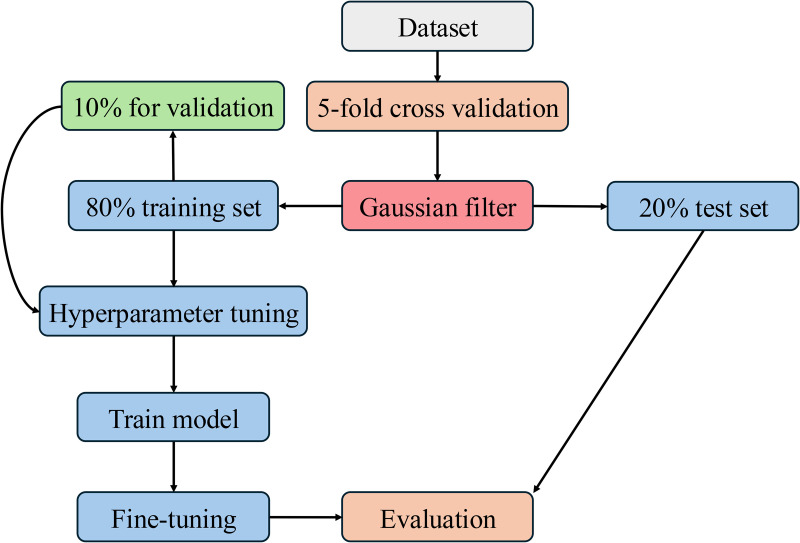
Training and evaluation approach: The dataset is split into a training set and a test set. The Gaussian filter is applied to the training and test set, followed by hyperparameter tuning using transfer learning. The model with the best hyperparameters is trained and then fine-tuned. Finally, the resulting model is evaluated on the test set.

The entire training and evaluation process is shown in Fig 3. In addition, the complete process was conducted with and without the proposed preprocessing filter to assess the sensitivity of the model performance to this preprocessing step. Stratified 5-fold cross-validation was used as an internal robustness check to reduce the sensitivity of the reported performance to a single train-test split. The dataset is divided into five folds of equal size. For each cross-validation iteration, one fold was used as the test set and the remaining four folds were used as the train set. Thus, in each iteration, 80% of the data were available for training and validation, while 20% were held out for testing. The test fold was rotated across the five iterations, ensuring that each sample served as test data exactly once. Importantly, the test fold of a given iteration was not used for training, validation, or hyperparameter optimization. Within the 80% train set of each iteration, 10% was reserved as a validation set for hyperparameter optimization and early stopping. Therefore, relative to the complete dataset, each iteration used approximately 72% of the data for training, 8% for validation, and 20% for testing. The validation set was always sampled only from the train set of the respective fold. On average, each class in the training set includes 403 images. To ensure the reproducibility of the method, we set a random seed of 42. Runs with other random seeds were not performed. Then we use the random search tuner of the Keras library with the aim of minimizing the sparse categorical cross-entropy loss on the validation set. The tuner is used to optimize the hyperparameters of the model and uses the validation split for evaluation. The tuner has a maximum number of 20 trials. Hyperparameter tuning is performed independently within each fold using the corresponding training and validation split. This ensures that no information from the test fold is used during model selection and prevents data leakage across folds. However, this approach increases computational cost as the tuning process is repeated for each fold. Since the same validation split is used within each fold for both hyperparameter optimization and early stopping, repeated evaluation may increase the risk of overfitting to the validation data. Early stopping from the Keras callbacks module (keras.callbacks.EarlyStopping with monitor = ’val_loss’, ’mode’ = min, patience = 10, min_delta = 1e-4, restore_best_weights = True) was applied during tuning and the subsequent final training, terminating whenever the validation loss failed to decrease for ten consecutive epochs. Each trial is conducted with 40 epochs. Throughout the optimization process, data augmentation techniques such as random rotation, random zoom, and random translation (height and weight) are applied to increase the variance of the dataset. No data augmentation was applied to the validation set or test set, it was applied only to the training data. Within each trial, the model weights corresponding to the lowest validation loss are restored. The best hyperparameter configuration is selected based on the lowest validation loss. The parameters that are optimized by the tuner and the corresponding values are shown in Table 2. During optimization, three optimizers are available: Adam, SGD, and RMSprop. The default values are used for each of these.

**Table 2:** Overview of the hyperparameters optimized during random search, including their search intervals, step sizes, and categorical choices.

Hyperparameter	Minimum Value	Maximum Value	Step
Random Rotation	0.05	0.2	0.05
Random Zoom	0.05	0.2	0.05
Random Translation Height	0.05	0.2	0.05
Random Translation Width	0.05	0.2	0.05
Dimensionality of first Dense layer	64	128	32
Dropout Rate	0	0.3	0.1
Learning Rate (Choices)	10^−5^	10^−4^	10^−3^
Optimizer	–	–	Adam, SGD, RMSprop

The best hyperparameters are then used to train the model. Only the custom layers are trained while the base model remains frozen. Training runs for up to 100 epochs with a batch size of 16. If the validation loss does not decrease for ten epochs, training is stopped by the early stopping callback and the best weights are restored. Afterwards, the base model is unfrozen and fine-tuned for up to 30 epochs with a learning rate of 10^−5^ and early stopping (patience = 10). The best model is again restored for evaluation. The model is trained independently in each fold using the corresponding training and validation subsets and is then evaluated on the respective 20% test fold, which is held out during training and hyperparameter tuning. Final performance is reported as the average across the five test folds.

### Evaluation metrics

To evaluate and interpret the model’s performance, we employ the following performance indicators: accuracy, balanced accuracy, True positive rate (TPR, sensitivity or recall), True negative rate (TNR, specificity), Positive predictive value (PPV, precision), Negative predictive value (NPV), Cohen’s Kappa, and F1-score. The accuracy determines the overall effectiveness of a model [35].However, it should be interpreted alongside complementary metrics, as aggregate accuracy may conceal class-specific performance differences in multiclass classification, particularly when there are class imbalances [36]. The following metrics are defined for the multi-class setting and incorporate class weighting [37,38]. In this context, $n_{i}$ indicates the number of samples in class *S* represents the total number of samples. To mitigate inflated performance estimates on imbalanced datasets, balanced accuracy can be utilized. For multiclass problems, such as the one considered in this study, balanced accuracy is calculated as the unweighted arithmetic mean of the class-wise TPRs [37]. *N* denotes the number of classes. In the multiclass setting, class-wise TPR, TNR, PPV, NPV and F1-score are first computed in a one-vs-rest manner for each class and are then averaged across classes using the corresponding class proportions. The TPR metric indicates the proportion of correctly identified samples of a given class, and maximizing it increases the likelihood of correctly identifying true members of that class [39]. The TNR, on the other hand, indicates how effectively a classifier identifies samples that do not belong to a given class in a one-vs-rest setting [35]. The PPV metric assesses prediction accuracy for a given class by indicating the proportion of class-specific positive predictions that correctly match true positive instances [39]. The NPV is calculated in the same one-vs-rest setting and represents the ratio of correctly classified negative samples to all samples classified as negative for a given class [40]. Cohen’s Kappa describes the reliability of a model by measuring the agreement between two judgments. It ranges from −1–1, where a Cohen’s Kappa of −1 indicates complete disagreement, and a Cohen’s Kappa of 1 signifies perfect agreement [41]. The harmonic mean of precision and recall, called F1-score, ranges from 0 to 1, with the minimum (0) occurring when all positive samples are misclassified (true positives = 0) and the maximum (1) occurring for perfect classification (false negatives = false positives = 0) [42]. The formula used for the multiclass case is provided by [43].


$$\begin{array}{c} \text{Accuracy}=\frac{TP+TN}{TP+TN+FP+FN} \end{array}$$ (2)

$$\begin{array}{c} \text{Balanced Accuracy}=\frac{1}{N}\sum_{i=1}^{N}\frac{TP_{i}}{TP_{i}+FN_{i}} \end{array}$$ (3)

$$\begin{array}{c} \text{TPR}=\sum_{i=1}^{N}\frac{n_{i}}{S}\cdot \frac{TP_{i}}{TP_{i}+FN_{i}} \end{array}$$ (4)

$$\begin{array}{c} \text{PPV}=\sum_{i=1}^{N}\frac{n_{i}}{S}\cdot \frac{TP_{i}}{TP_{i}+FP_{i}} \end{array}$$ (5)

$$\begin{array}{c} \text{NPV}=\sum_{i=1}^{N}\frac{n_{i}}{S}\cdot \frac{TN_{i}}{TN_{i}+FN_{i}} \end{array}$$ (6)

$$\begin{array}{c} \text{TNR}=\sum_{i=1}^{N}\frac{n_{i}}{S}\cdot \frac{TN_{i}}{TN_{i}+FP_{i}} \end{array}$$ (7)

$$\begin{array}{c} \text{Cohen's Kappa}=\frac{P_{0}-P_{e}}{1-P_{e}} \end{array}$$ (8)

$$\begin{array}{c} \text{F1-Score}=\sum_{i=1}^{N}\frac{n_{i}}{S}\;2\cdot \frac{PPV_{i}\cdot TPR_{i}}{PPV_{i}+TPR_{i}} \end{array}$$ (9)

TP = True Positives

TN = True Negatives

FP = False Positives

FN = False Negatives

P_0_ = Observed Agreement

P_*e*_ = Expected Agreement

### Dataset

This study utilizes the “Image Dataset for Radish Plant Leaf Disease Detection and Freshness Assessment from Bangladesh” [1] (https://doi.org/10.17632/s973cz2jcd.1), which comprises 5 classes and a total of 2,801 color images of radish plant leaves, with images in 700×700 px jpg format. The images do not contain any visible annotations. Table 3 presents four different properties of the dataset images to illustrate the model’s within datase robustness to variations. The signal-to-noise ratio was computed by estimating noise with the Immerkaer method and expressing it relative to the signal [44]. For colorfulness, we followed the methodology of Hasler and Suesstrunk [45]. The dataset is divided into 2,188 images of radish plant leaf diseases and 613 images of healthy radish plant leaves. The 2,188 images of radish plant leaf diseases are categorized in 4 classes: Radish Black leaf spot (526), Radish Downy mildew (601), Radish flea beetle (513), and Radish Mosaic virus (548). The images depicting the healthy radish plant leaves are categorized under the class Radish Fresh leaf (613). The training set exclusively contains comprehensive images without visible artifacts unrelated to the respective class. On average, each class in the training set includes 403 images. The folder names in the test dataset indicate the type of class, with the numbers in parentheses specifying the quantity of images available for each category.

**Table 3:** Image-property statistics of the dataset, including brightness, signal-to-noise ratio, saturation, and colorfulness. Values are reported as mean and standard deviation.

Property	Value
Brightness	Mean: 0.2609; Std. dev.: 0.0441
Signal to noise ratio (Immerkaer method)	Mean: 17.3950 dB; Std. dev.: 2.7720 dB
Saturation	Mean: 0.1423; Std. dev.: 0.0657
Colorfulness	Mean: 0.1133; Std. dev.: 0.0362

### Setup

For training and testing the architecture an NVIDIA A100 PCIe GPU with 40GB memory is used. The experiments were conducted using TensorFlow (2.19.0), Keras (3.10.0), Python 3.11.7, and CUDA 12.4.1. Random Search, provided by the Keras-Tuner package (version 1.4.6), was employed to identify the optimal parameters. To avoid overfitting and save computation time, the callback function *early stopping* was used, which stops the training after 10 consecutive epochs in which the validation loss has not decreased. Scikit-learn (version 1.7.0) was utilized for stratified cross-validation and the computation of performance indicators. Throughout the entire training and validation process, the images were converted to a resolution of 150×150 pixels.

## Results

Our novel deep learning approach was evaluated using stratified 5-fold cross-validation. The average values of the performance metrics from 5 folds with and without an applied preprocessing filter are presented in Table 4.

**Table 4:** Comparison of average evaluation metrics for the classification model with and without Gaussian filter, based on stratified 5-fold cross-validation. For each metric, the superior result is typeset in bold.

Performance indicator	Without Gaussian filter [percent]	With Gaussian filter [percent]
Accuracy	99.22	**99.86**
Balanced Accuracy	99.23	**99.86**
True Positive Rate	99.22	**99.86**
True Negative Rate	99.81	**99.96**
Positive Predictive Value	99.22	**99.86**
Negative Predictive Value	99.80	**99.96**
Cohen’s Kappa	99.02	**99.82**
F1-Score	99.22	**99.86**

Each pre-trained architecture was assessed in terms of overall accuracy, class-averaged TPR, PPV, and Cohen’s Kappa. In addition, we report the F1-score, balanced accuracy, TNR, and NPV to provide additional information on classification performance. Additional significance tests were conducted to assess whether the model with Gaussian filter achieved significantly higher balanced accuracy than the model without Gaussian filter. For this purpose, the balanced accuracy values of the five folds were compared. The model with Gaussian filter achieved balanced accuracy values of 99.83% in Fold 1, 99.84% in Fold 2, 100% in Fold 3, 100% in Fold 4, and 99.65% in Fold 5. The corresponding values for the model without Gaussian filter were 99.46% in Fold 1, 98.72% in Fold 2, 99.16% in Fold 3, 99.67% in Fold 4, and 99.12% in Fold 5. Since the comparison is based on paired fold-wise results, a paired t-test and a Wilcoxon signed-rank test were applied with the alternative hypothesis that the model with Gaussian filter performs better than the model without Gaussian filter. The paired t-test yielded $p_{\text{t-test}}=0.0066$ and the Wilcoxon signed-rank test yielded *p*_Wilcoxon_ = 0.0313. Both p-values are below the significance level of 0.05, indicating that the model with Gaussian filter achieved significantly higher balanced accuracy than the model without Gaussian filter.

To compare the advantage of using the VGG16 architecture, Table 5 includes five additional architectures, each reported with its achieved balanced accuracy, required training time, and test-time per image. It can be observed that VGG16 not only achieves the highest balanced accuracy and the shortest training time, but also yields a very low test-time per image. High balanced accuracy is particularly crucial for the detection of plant diseases, which is why this architecture was chosen.

**Table 5:** Comparison of balanced accuracy, training time, and test time per image for six architectures, including the architecture used. Bold values indicate the best value.

	Balanced Accuracy (%)	Training time	Mean test time per image (ms)
ResNet50	94.57	1h 19m 21s	60.68
VGG19	98.81	1h 26m 9s	**55.45**
Inception V3	97.06	1h 26m 5s	63.01
Xception	98.80	1h 24m 48s	59.07
EfficientNetB0	98.53	1h 36m 11s	60.67
VGG16	**99.23**	**1h 16m 26s**	55.66

As shown in Table 4, the application of Gaussian filtering with σ=0 and a 7×7 kernel led to a consistent improvement across all performance indicators. Compared to the version without preprocessing, accuracy increased by 0.64 pp, and balanced accuracy rose by 0.63 pp. Sensitivity and F1-score each improved by 0.64 pp. Specificity also increased slightly, by 0.15 pp. Both the PPV and NPV increased by 0.64 and 0.16 pp, respectively. The largest improvement was observed in Cohen’s Kappa, which increased by 0.80 pp. In terms of accuracy, the application of the Gaussian filter resulted in an increase of 0.64 pp, which corresponds to a reduction in errors of 82.05% ($Error\;rate=\frac{Error_{A}-Error_{B}}{Error_{A}}$). Compared to the previous benchmark of 94.37% [30], our approach achieves a 5.49% better result, which in relative terms leads to a 97.51% reduction in errors.

Table 6 summarizes the model’s performance with Gaussian filtering across all five folds of the stratified cross-validation. In addition, Table 7 shows the individual TPR values for each class and each fold.

**Table 6:** Results of the evaluated performance indicators for the proposed approach with Gaussian filtering across the five cross-validation folds. The AVG column reports the mean value across all folds.

Metric\Fold	1	2	3	4	5	AVG
Accuracy	99.82	99.82	100	100	99.64	**99.86**
Balanced Accuracy	99.83	99.84	100	100	99.65	**99.86**
True Positive Rate	99.82	99.82	100	100	99.64	**99.86**
True Negative Rate	99.96	99.96	100	100	99.91	**99.96**
Positive Predictive Value	99.82	99.82	100	100	99.64	**99.86**
Negative Predictive Value	99.95	99.95	100	100	99.91	**99.96**
Cohen’s Kappa	0.9978	0.9978	1	1	0.9955	**0.9982**
F1-Score	99.82	99.82	100	100	99.64	**99.86**

**Table 7:** TPR results (in %) with average value and standard deviation by class for the five runs of stratified cross-validation.

Class\Fold	1	2	3	4	5	AVG + Std. Dev.
Black leaf spot	100	100	100	100	100	**100 ± 0.00**
Downy mildew	99.17	100	100	100	100	**99.83 ± 0.37**
Fresh leaf	100	99.18	100	100	99.19	**99.67 ± 0.45**
Mosaic virus	100	100	100	100	99.08	**99.82 ± 0.41**
Flea beetle	100	100	100	100	100	**100 ± 0.00**

**Fig 4:**
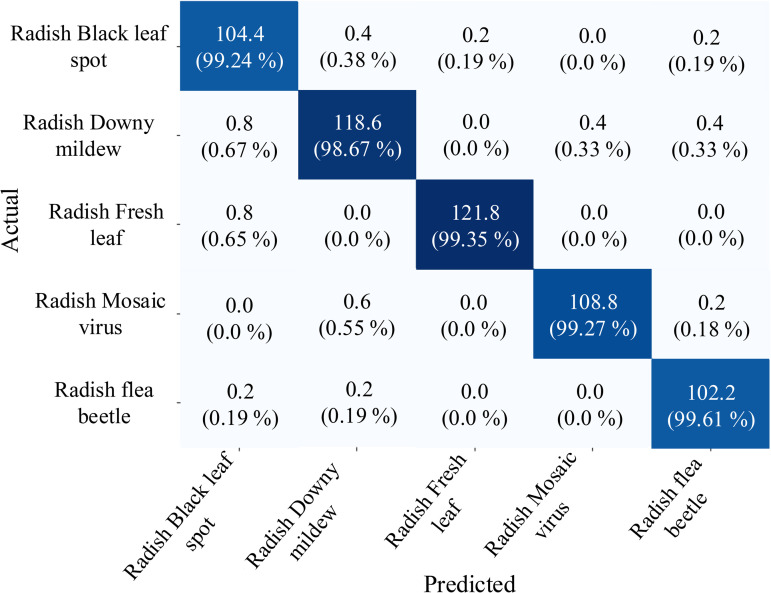
Average confusion matrix across the five folds without applied Gaussian filtering. Rows indicate the actual classes, columns indicate the predicted classes, and cell values represent averaged counts over the five folds, with percentages shown in parentheses.

**Fig 5:**
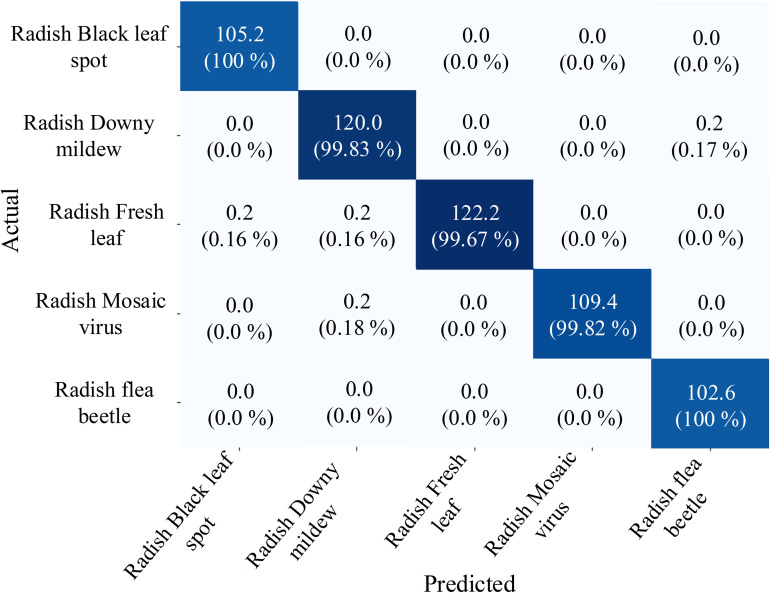
Average confusion matrix across the five folds with applied Gaussian filtering. Rows indicate the actual classes, columns indicate the predicted classes, and cell values represent averaged counts over the five folds, with percentages shown in parentheses.

The results show strong consistency: accuracy ranged from 99.64% to 100%, and balanced accuracy from 99.65% to 100%. TPR, PPV, and F1-score fluctuated by at most 0.36 pp. TNR and NPV remained stable between 99.91% and 100%. The largest variation was observed in Cohen’s Kappa (0.9955–1), though values stayed consistently high. Balanced accuracy and the reported TPR are numerically very close because both are derived from class-wise TPR values but use different averaging schemes. Balanced Accuracy is unweighted, while the reported TPR is weighted by class size. Due to similar class sizes and consistently high class-wise TPRs, both values can appear identical after rounding. Similarly, the PPV and F1-score are comparable, as precision and recall at the class level are consistently high and closely related. The averaged confusion matrices in Figs 4 and 5 highlight class-wise improvements after applying the Gaussian filter. The confusion matrices present averaged counts over five folds. For Radish Black leaf spot, accuracy increased from 104.4 (99.24%) to 105.2 (100%), eliminating misclassifications into Downy mildew, Fresh leaf, and flea beetle. Radish Downy mildew improved from 118.6 (98.67%) to 120.0 (99.83%), removing confusion with Black leaf spot and Mosaic virus, and reducing errors into flea beetle. Radish Fresh leaf rose from 121.8 (99.35%) to 122.2 (99.67%), with fewer errors, mostly toward Downy mildew. Radish Mosaic virus increased from 108.8 (99.27%) to 109.4 (99.82%), eliminating confusion with flea beetle and reducing errors into Downy mildew. Radish flea beetle improved from 102.2 (99.61%) to 102.6 (100%), removing all prior misclassifications into Black leaf spot and Downy mildew.

**Fig 6:**
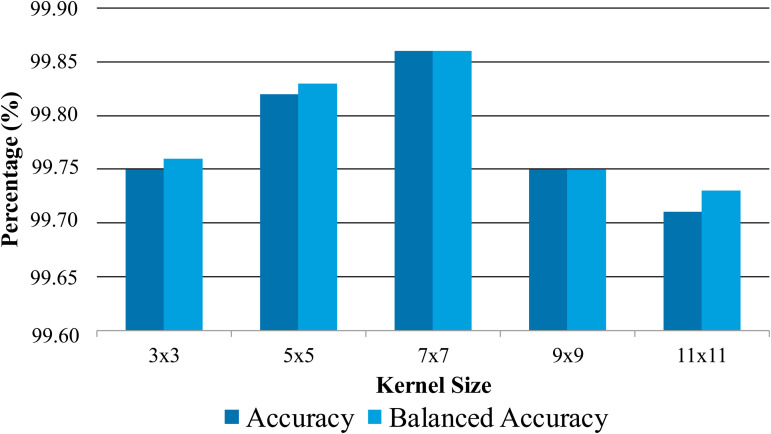
Graphical demonstration of the impact of Gaussian filter kernel size on accuracy and balanced accuracy.

**Fig 7:**
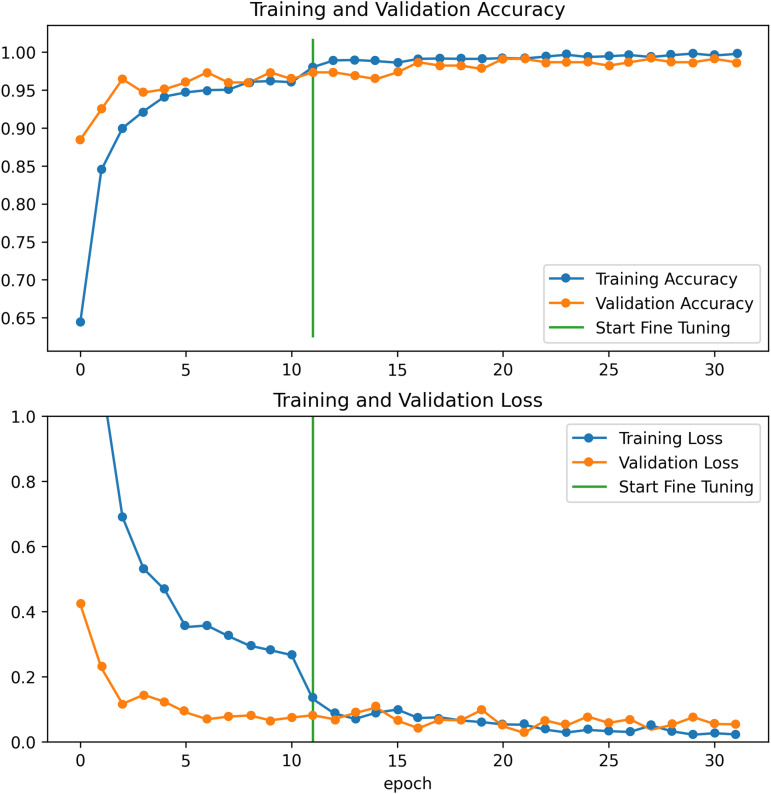
Training and validation accuracy and loss curves of the proposed deep-learning model without applied Gaussian filtering. The upper panel shows accuracy across epochs, while the lower panel shows the corresponding loss values. The vertical green line marks the start of fine-tuning.

**Fig 8:**
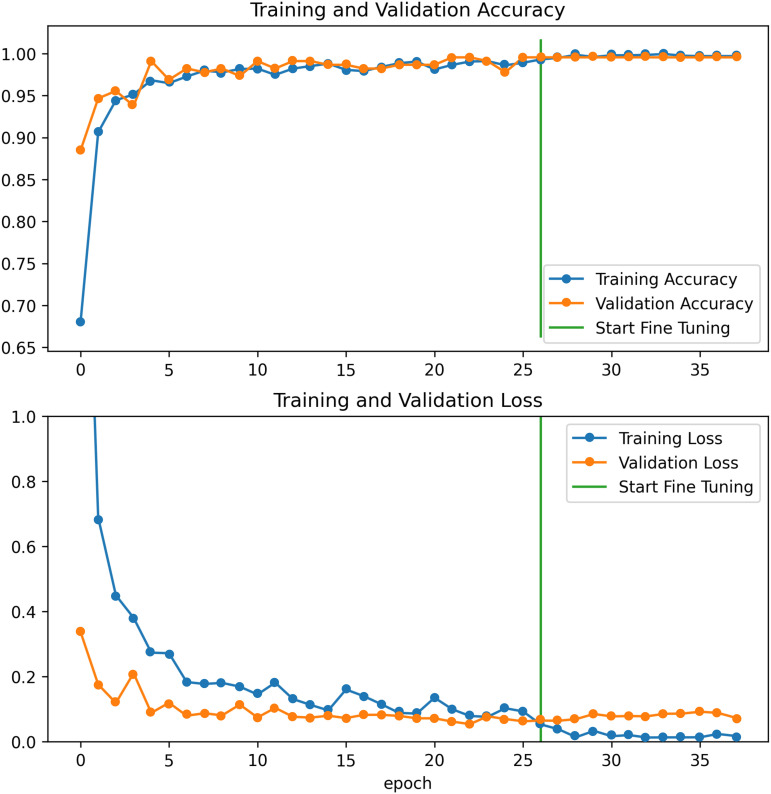
Training and validation accuracy and loss curves of the proposed deep-learning model with applied Gaussian filtering. The upper panel shows accuracy across epochs, while the lower panel shows the corresponding loss values. The vertical green line marks the start of fine-tuning.

The average class-wise classification results over five folds with applied Gaussian filter are presented in Fig 5 as percentages. Radish Black leaf spot images were classified with 100% accuracy. For Radish Downy mildew, 0.17% of the images were misclassified as Radish flea beetle. In the case of Radish Fresh leaf, 0.16% were incorrectly classified as Radish Black leaf spot and another 0.16% as Radish Downy mildew. For Radish Mosaic virus, 0.18% were misclassified as Radish Downy mildew. No misclassifications occurred for Radish flea beetle, which was classified with 100% accuracy. Fig 6 illustrates the impact of different Gaussian kernel sizes on the accuracy and balanced accuracy metrics. Among the tested configurations, the 7×7 kernel yielded the highest values for both metrics. In addition, further preprocessing filters and methods were tested, as shown in Table 8, including the median filter, the bilateral filter, contrast normalization using Contrast Limited Adaptive Histogram Equalization (CLAHE), and gray-world color correction. Each of these was tested with different parameter settings, the best result in terms of balanced accuracy was included in the table using the optimal parameters. It appears that the Gaussian filter achieves the highest balanced accuracy Figs 7 and 8 show the training and validation curves of the proposed model without and with the Gaussian preprocessing filter, respectively. Several consistent differences can be observed. Fig 8 shows that the training loss curve is smoother and exhibits fewer fluctuations compared to the model without preprocessing. The validation loss also follows a more stable trajectory throughout the training epochs. The transition to fine-tuning occurs later, and the gap between training and validation loss is slightly reduced. Similarly, the training and validation accuracy curves display fewer fluctuations and remain closely aligned over time.

**Table 8:** Evaluation of additional preprocessing filters compared to the results of the Gaussian filter. The best result in terms of balanced accuracy is shown. Each filter was tested with five parameter settings: median filter (kernel sizes: 3, 5, 7, 9, 11), bilateral filter (diameter: 3, 5, 7, 9, 11), contrast normalization (CLAHE) (clip limit: 0.5, 1, 2, 3, 4) and gray-world color correction (strength of color correction: 0.2, 0.4, 0.6, 0.8, 1.0).

	Configuration	Balanced Accuracy (%)
Gaussian Filter	kernel size = 7	99.86
Median Filter	kernel size = 5	99.59
Bilateral Filter	diameter = 9	99.44
Contrast Normalization	clip limit = 0.5	99.32
Color Correction	correction strength = 0.4	99.52

## Discussion

### Model performance analysis and limitations

The proposed approach, which integrates a Gaussian preprocessing filter, led to consistent improvements across all evaluated performance indicators. Compared to the model without preprocessing, measurable gains were observed in accuracy, balanced accuracy, F1-score, and Cohen’s Kappa, metrics that reflect both overall classification reliability and class-wise consistency. The averaged results with and without the applied Gaussian filter across five folds are reported in Table 4, demonstrating a clear performance advantage attributable to the filtering step. Contextually compared to the previous benchmark in [30], our approach achieves an improvement in accuracy of 5.49 percentage points. This increase in accuracy also results in a reduction in the error rate. A lower error rate is particularly relevant in plant disease recognition, as it decreases the risk of undetected infections that may lead to yield losses and reduces false alarms, thereby preventing unnecessary pesticide applications. In the context of the dataset used in this study, this corresponds to a relative reduction of 97.51% in the error rate compared to the benchmark reported in [30]. These findings suggest that incorporating image preprocessing into deep learning pipelines can meaningfully improve model classification performance and performance consistency. The observed performance enhancement may reflect a beneficial preprocessing effect of the Gaussian filter, potentially by reducing high-frequency visual variation while preserving the overall structure of disease-associated image patterns, such as lesion boundaries, vein patterns, and discoloration textures. Such disease indications can be subtle and localized and are therefore particularly important to highlight. As a result, they can benefit from the smoothing effect of Gaussian filtering.

As shown in the comparison between Fig 4 (without preprocessing) and Fig 5 (with Gaussian filter), classification performance improved across all classes following the application of the preprocessing filter. Fig 5 shows that misclassifications were rare and limited to biologically or visually adjacent categories. For example, 0.16% (1 sample each in absolute numbers) of Radish Fresh leaf samples were misclassified as either Radish Black leaf spot or Radish Downy mildew. Upon examination of the fresh sample, small dark spots are visible on the leaf surface, likely caused by contamination. Although these irregularly shaped discolorations are less pronounced than those seen in black leaf spot, they have a similar local structure and could therefore be the reason for the misclassification. In the classification of the second fresh sample as Downy mildew, specular highlights may have played a larger role, as they can appear similar to disease-like brightened areas. Similarly, 0.18 percent of the images (in absolute numbers 1 sample) infected with the radish mosaic virus were incorrectly classified as radish downy mildew, likely due to diffuse yellow-green discoloration and pale leaf areas that visually overlap with the symptoms of downy mildew. It is possible that the mosaic pattern in the misclassified sample is not particularly pronounced, so the model may have focused more on local discoloration and small surface features that resembled downy mildew. The sample with downy mildew may have been mistakenly classified as radish flea beetle damage, as it shows distinct tissue damage and also has several holes, which are more typical of flea beetles. The model may therefore have focused more on these defects than on other characteristics associated with downy mildew. Notably, Radish Black leaf spot and Radish flea beetle were both classified with 100% accuracy, likely due to their pronounced and distinct visual signatures, such as sharply defined necrotic areas or characteristic hole patterns. These improvements may be explained by the smoothing effect of the Gaussian filter, which may have mitigated irrelevant visual detail while simultaneously enhancing the visibility of structurally relevant features. This selective amplification of key features could have facilitated more accurate class separation. Overall, the Gaussian filter appears to have enhanced intra-class consistency while reducing inter-class confusion.

Table 5 compares the effectiveness of the selected architecture with that of other architectures. VGG16 achieves the highest balanced accuracy and the lowest training time. We hypothesize that these results may be due to a variety of reasons. A key feature of this architecture is its exclusive use of 3×3 convolutional layers, which may excel in capturing small details. Additionally, spatial dimensions are reduced late in the process using max pooling so fine structures are preserved longer. ResNet50 consists of 50 layers organized into four components: convolutional layers, identity blocks, convolutional blocks, and fully connected layers. ResNet50 features identity mapping through identity blocks, which allow certain layers to be bypassed when unnecessary. However, skip connections could be problematic because they might shorten the learning path. This shorter path may cause crucial information, such as subtle details on the leaves, to be ignored or given too little weight. Furthermore, unlike VGG16, downsampling occurs early on, which could lead to a loss of information. VGG19 has a structure similar to VGG16 but has more convolutional layers, which could cause it to focus more on unimportant areas than important ones. InceptionV3 relies on three Inception modules. The idea is to apply different filters with various kernel sizes to the same layer through convolution. These paths, each with different filter sizes, run in parallel until they are concatenated at the end. After the first two Inception modules, a grid size reduction block follows to reduce the spatial dimension. In general, InceptionV3 quickly and significantly reduces the spatial dimension compared to VGG16. This could cause important and subtle disease information to be lost from the leaves. Furthermore, the individual paths in the inception blocks are concatenated, and an unweighted fusion of the features could contribute to important information being overlooked, thus distorting the result. Xception is conceptually related to InceptionV3 but replaces its modules with Depthwise Separable Conv blocks constructed serially and connected by residual connections. The potential problem of unweighted fusion, as in InceptionV3, is eliminated. However, due to the skip connections used in Xception, the same potential disadvantages exist due to a shortened learning path. EfficientNetB0 uses 16 Mobile Inverted Bottleneck Convolution (MBConv) blocks, combining depthwise separable convolutions with squeeze-and-excitation optimization. Unlike VGG16, 5×5 layers are used in addition to 3×3 convolutional layers. Larger kernels may be less effective at capturing smaller structures, and when they do, the structures are captured in a weakened form. The downstream squeeze-and-excitation block amplifies these larger details and further weakens the smaller ones. This could lead to smaller disease features being suppressed.

The observed training dynamics further (Fig 8) highlight the benefit of Gaussian preprocessing. The proposed approach trained with the filter exhibited smoother loss curves, reduced variance between training and validation accuracy, and a delayed yet more stable transition from feature reuse to fine-tuning. These effects suggest improved convergence stability and reduced overfitting risk. By attenuating high-frequency noise in the input images, the Gaussian filter likely contributed to a smoother loss landscape. This may have facilitated more stable gradients during optimization, particularly in the early training stages, allowing the model to generalize more effectively from the frozen feature representations. The result is a training process that is likely both more stable and more efficient, especially under limited data conditions. Compared to the training without Gaussian filtering (Fig 7), fine-tuning (green vertical line) begins at a later stage. This delay arises because the blur operation smooths the inputs and attenuates edges, thereby reducing the local variations in the data. As a result, the gradients during optimization tend to be smaller and more uniform, which stabilizes the overall learning dynamics. However, this has the side effect of slowing down convergence and requiring the model to undergo more training epochs in order to learn the features from the images. In real-world agricultural scenarios, especially field monitoring and supply-chain quality assurance, several metrics are important for interpreting the model’s performance. High TPR supports the early detection of infected leaves and can enable timely intervention, while high PPV limits false positives, helping to reduce unnecessary pesticide use and preserve crop integrity. Balanced accuracy is particularly relevant for imbalanced multiclass datasets, as it reflects average class-wise recall and helps ensure that less frequent but harmful classes are not overlooked. TNR and NPV provide additional information in this study, but they are calculated in a one-vs-rest manner and should therefore be interpreted more cautiously than in binary classification. In this context, high TNR indicates that samples from other classes are rarely assigned to a given class, while high NPV indicates that samples predicted as not belonging to a given class generally do not belong to that class. As Table 6 shows, our model attains strong values across these metrics, with the most informative multiclass indicators being balanced accuracy, TPR, PPV, and F1-score. These results indicate reliable classification performance and may support applications aimed at reducing crop loss, stabilizing yield, and improving food security and supply-chain efficiency.

As Fig 6 indicates, Gaussian kernel size notably influences accuracy and balanced accuracy, a 7×7 kernel delivered the best overall performance. The tested kernel sizes (3×3–11×11) were chosen to compare different levels of smoothing, from weak to stronger blurring. Larger kernel sizes were not considered, as excessive smoothing may remove small disease-related details. The standard deviation σ was set to 0, where σ is derived from the kernel size. This avoids introducing an additional independent parameter and allows the effect of smoothing to be analyzed in a controlled and interpretable way based solely on the kernel size. Hence, kernel size should be treated as a tunable hyperparameter rather than a fixed preprocessing choice. The non-linear relation between kernel size and performance underscores that even low-level preprocessing decisions can substantially affect classification quality. Carefully chosen filtering can improve accuracy without increasing model complexity, showing that gains are achievable beyond architectural changes.

Table 3 shows that the real-world radish dataset [1] exhibits a signal-to-noise ratio of 17.3950 dB with a standard deviation of 2.7720 dB, indicating that our model was trained on data with varying noise levels. The mean brightness of 0.2609 (standard deviation: 0.0441) further reflects differences in illumination conditions. The mean saturation of 0.1423 indicates generally low color saturation. However, the standard deviation of 0.0657 (46.17%) suggests substantial variability across images. Similarly, the mean colorfulness of 0.1133 points to overall modest colorfulness, while its standard deviation of 0.0362 (31.95%) again indicates high diversity within the dataset. The characteristics of the dataset, with its varying image conditions, provide a basis for evaluating model performance under intra-dataset variability. Together with stratified 5-fold cross-validation for evaluation, these characteristics demonstrate that the proposed approach already achieves strong performance consistency within the evaluated dataset. Nevertheless, comparability with other datasets may be limited. The dataset used in this study comprises five classes, and for other radish datasets that include different disease categories, the model’s performance may vary. Although the present study focuses on radish leaf diseases, the results suggest that the proposed approach may be a promising basis for future studies on other plant species. However, cross-dataset validation is required before conclusions about transferability to other crops can be drawn.

### Contextual comparison with related work and methodological contributions

A contextual comparison with the related work, by Quoc et al. [30], Banerjee et al. [26], and Ji et al. [27], highlights the methodological and empirical strengths of our proposed approach. Quoc et al. [30] proposed SCOLD, which reached 95.82% in a 16-shot few-shot setting and 94.37% accuracy after fine-tuning. No further classification metrics were provided for radish leaf disease performance. Banerjee et al. [26] reported an accuracy of 92.00% and a weighted average F1-score of 81.45% using a CNN–SVM hybrid model. Precision and recall were reported at the class level, ranging from 76.19% to 81.82% and from 75.00% to 82.05%, respectively. However, no values for balanced accuracy, TNR, or NPV were reported. Ji et al. [27] achieved an accuracy of 91.00%, with reported values of 93.00% for precision, 89.00% for recall, and a mean average precision (mAP) of 90.00% with the proposed approach. However, their work did not report F1-score, TNR, or balanced accuracy. In contrast, our study reports a broader number of performance indicators. These metrics provide additional insight into different aspects of the model’s classification performance. Recall and F1-score are particularly relevant in field monitoring, ensuring robust and reliable identification of diseased leaves. Precision helps reduce false alarms and can support more targeted pesticide use. Balanced accuracy safeguards against bias toward dominant classes by reflecting the average recall across all classes. TNR and NPV are included as supplementary one-vs-rest metrics and provide additional information on class-wise exclusion behavior, while Cohen’s Kappa supports the assessment of overall agreement beyond chance. Altogether, the results suggest that our model could be a promising approach for further testing in in-field diagnostics and supply chain quality assurance.

Moreover, our study is the only one among the compared works to implement a 5-fold cross-validation scheme, which strengthens the reliability of the performance estimates and mitigates bias arising from sample variability. This is particularly important for agricultural datasets characterized by heterogeneous leaf appearances and intra-class variability. Furthermore, we rely on the peer-reviewed, documented and publicly available dataset, ensuring transparency, reproducibility, and comparability of results. While this dataset was also used by Quoc et al. [30], it was not employed by Banerjee et al. [26] or Ji et al. [27], whose data sources were either self-assembled or insufficiently documented. This distinction is important, as the use of standardized and curated, publicly available datasets facilitates methodological reproducibility and strengthens the credibility of performance claims. Taken together, these results suggest that our approach achieves strong performance in radish plant disease classification. This is supported by multiple performance indicators and 5-fold cross-validation.

### Possible applications

Given the strong performance of the proposed model in classifying radish leaf diseases, it has considerable potential for practical deployment in agricultural regions where radish is widely cultivated. By providing a promising foundation for future applications which enable automated detection of leaf diseases – via drones, tractor-mounted cameras, or stationary imaging systems – the model could help prevent yield losses and reduce damage through timely intervention. This may lead to cost savings, more efficient use of resources, and improved crop health. The model could also be a methodological base for future applications that support quality control in the post-harvest supply chain, allowing infected or damaged produce to be identified and removed before distribution. This may reduce post-harvest losses and contribute to improved product quality and market value. In addition, the model could be integrated into future applications for digital farming platforms and precision agriculture systems, enabling real-time disease diagnostics within broader farm management tools. This would not only save time and labor in large-scale operations but also promote sustainable agricultural practices by enabling more targeted use of agrochemicals and reducing unnecessary pesticide application. Importantly, such tools could be particularly beneficial in regions with limited access to agronomic expertise, helping smallholder farmers detect diseases early and bridge knowledge gaps. By using (or advancing) this approach, users could also contribute to Goal 2 of the UN Sustainable Development Goals by identifying and treating diseased plants in the field or, if necessary, destroying them to prevent the spread of disease [46]. Although this would initially lead to a reduction in stock, it could prevent further plants from becoming diseased, which could have led to greater losses in the long term, and could therefore lead to higher crop yields and thus greater food security overall. It could also enable more sustainable agriculture to be achieved. Furthermore, the use of this approach in the supply chain as part of quality assurance could contribute to Goal 12 of the UN Sustainable Development Goals by identifying diseased products and disposing of only those, which could lead to reduced waste [46]. Similarly, in future applications, diseased food could be identified and destroyed in the supply chain, preventing it from spreading to other food post-harvest and, mitigating food loss and, thus enable sustainable consumption. Altogether, the model may provide a methodological basis for future applications aimed at economic efficiency, environmental sustainability, and improved food security, pending successful field validation and implementation.

## Conclusion

Deep learning continues to show great promise for plant disease detection and agricultural quality assurance. We identified potential for improvement in the classification of diseases on radish plants and addressed it by developing an effective deep-learning approach designed to capture fine-grained local patterns. Gaussian filtering was applied as a preprocessing step to potentially reduce high-frequency visual variation and contribute to the improved performance observed in the evaluated classification setting. The proposed approach was evaluated on a publicly available and documented dataset from a peer-reviewed source using stratified 5-fold cross-validation. The results demonstrate that our method consistently achieved high performance across all metrics, including an average accuracy and balanced accuracy of 99.86%. This sets a new dataset-level benchmark in this problem domain. Our study not only presents an novel deep learning approach that combines an established CNN with an established preprocessing filter for domain-specific challenges in plant leaf disease classification and establishes a new performance standard, but also shows that a carefully chosen preprocessing filter can achieve high classification accuracy without increasing model complexity. Furthermore, the proposed approach has potential being a methodological foundation for a wide range of future operational applications, including field monitoring, yield protection, and post-harvest quality control. By enabling robust and accurate detection of diseases, it contributes to improved food security, reduced economic losses, and more sustainable agricultural practices.

### Limitations

Despite the promising results achieved by the proposed deep learning approach for radish leaf disease classification, several limitations must be acknowledged. The evaluation is restricted to the dataset provided, which contains four disease classes. Therefore, the model’s performance and reliability to other radish leaf diseases not represented in this dataset remain uncertain. Furthermore, potential sources of bias may influence the observed performance. Although all images were acquired under standardized conditions, dataset-specific biases may still arise from consistent imaging settings, lighting conditions, leaf positioning, or the number of disease stages. In addition, class-specific differences in visual distinguishability can lead to bias, as diseases with obvious visual characteristics are easier to classify than subtle symptoms or those that appear in the early stages. The interpretation of the model’s behavior – particularly the effect of the Gaussian filter applied during preprocessing – is constrained to standard evaluation metrics. Consequently, the underlying mechanisms beyond these metrics that contribute to the filter’s effectiveness remain unclear. Although stratified cross-validation provides a robust estimate of performance within the dataset, the absence of external validation using independent datasets means that the reported results primarily reflect performance within the specific application domain and may not be fully transferable to unseen real-world scenarios.

### Future work

To strengthen external validity and robustness, future work will evaluate the model on additional datasets and disease classes, enabling a more reliable assessment of performance across a broader spectrum of radish leaf diseases. Furthermore, we plan to evaluate the model on different crop species and its generalization. For future work, we plan to investigate additional architectural variants as well as further preprocessing methods to further improve performance. In addition, we plan to move from the current testing phase to the field phase in future work and evaluate the approach under real-world conditions.

## Supporting information

S1 File Supporting information files. (XLSX)
